# *Glaesserella parasuis* serotype 5 breaches the porcine respiratory epithelial barrier by inducing autophagy and blocking the cell membrane Claudin-1 replenishment

**DOI:** 10.1371/journal.ppat.1010912

**Published:** 2022-10-13

**Authors:** Mingxing Liu, Qing Wang, Wenda Wu, Min Chen, Pengyun Zhang, Mengru Guo, Huixing Lin, Zhe Ma, Hong Zhou, Hongjie Fan

**Affiliations:** 1 MOE Joint International Research Laboratory of Animal Health and Food Safety, College of Veterinary Medicine, Nanjing Agricultural University, Nanjing, China; 2 Anhui Province Key Laboratory of Veterinary Pathobiology and Disease Control, College of Animal Science and Technology, Anhui Agricultural University, Hefei, China; 3 Jiangsu Co-innovation Center for Prevention and Control of Important Animal Infectious Diseases and Zoonoses, Yangzhou, China; University of Illinois, UNITED STATES

## Abstract

*Glaesserella parasuis* (*G*. *parasuis*), the primary pathogen of Glässer’s disease, colonizes the upper respiratory tract and can break through the epithelial barrier of the respiratory tract, leading to lung infection. However, the underlying mechanisms for this adverse effect remain unclear. The *G*. *parasuis* serotype 5 SQ strain (HPS5-SQ) infection decreased the integrity of piglets’ lung Occludin and Claudin-1. Autophagy regulates the function of the epithelial barrier and tight junction proteins (TJs) expression. We tested the hypothesis that HPS5-SQ breaking through the porcine respiratory epithelial barrier was linked to autophagy and Claudin-1 degradation. When HPS5-SQ infected swine tracheal epithelial cells (STEC), autophagosomes encapsulated, and autolysosomes degraded oxidatively stressed mitochondria covered with Claudin-1. Furthermore, we found that autophagosomes encapsulating mitochondria resulted in cell membrane Claudin-1 being unable to be replenished after degradation and damaged the respiratory tract epithelial barrier. In conclusion, *G*. *parasuis* serotype 5 breaks through the porcine respiratory epithelial barrier by inducing autophagy and interrupting cell membrane Claudin-1 replenishment, clarifying the mechanism of the *G*. *parasuis* infection and providing a new potential target for drug design and vaccine development.

## Author summary

*G*. *parasuis* spreads in pig herd through respiratory transmission, which colonizes the porcine respiratory tract, damages porcine respiratory epithelial paracellular, and causes porcine lung damage. *G*. *parasuis* serotype 5 infection induces autophagy in porcine respiratory epithelial cells. Autophagy is a degradation pathway for cellular proteins, organelles, and invading pathogens. It has been reported that pathogens induce autophagic degradation of epithelial tight junction proteins to disrupt epithelial barrier tight junctions. Here we show that STEC Claudin-1 covering mitochondria is enveloped and degraded by autophagy in the infection of HPS5-SQ. Our work reveals the mechanism of Claudin-1 autophagic degradation cutting off the replenishment of cell membrane Claudin-1 and providing a specific target in anti-*G*. *parasuis* spread.

## Introduction

Swine Glässer’s disease is a worldwide epidemic, causing huge economic losses for the global swine industry. As the primary pathogen of Glässer’s disease, *Glaesserella parasuis* (*G*. *parasuis*) was firstly isolated in 1910, including at least 15 serotypes. Serotypes 5, 4, and 13 have a high isolation rate in sick swine lungs and are largely regarded as common pathogenic serotypes [1]. Especially, *G*. *parasuis* serotype 5 is considered the most virulent and has the potential to break through the epithelial barrier of the respiratory tract, resulting in severe lung infection [2, 3].

Tight junction proteins (TJs) are important components of the respiratory tract epithelial barrier and cell-cell connection structures [4, 5]. Claudin and Occludin family proteins are between adjacent cells, cylindrical cord structures formed by the plasma membrane of the tight junction region. These two family proteins connected cells with specific proteins and divalent cations to close the gap between cells [6]. Claudin-1 is a member of the claudin family proteins that plays a central role in tight junction structure. As an indispensable component of the paraepithelial barrier in vertebrates, the absence of Claudin-1 in the cell membrane is closely related to paraepithelial barrier damage [7]. In addition, the reduction of Claudin-1 in epithelial cell membranes caused by pathogens’ adhesion contributes to epithelial barrier damage and infection [8].

Autophagosomes encapsulate and confine the contents during the process of cellular Autophagy. Later, lysosomes and autophagosomes merge to break down the autophagosome contents [9]. When the body is severely infected, much ROS accumulates in the infected cells, causing an imbalance in cell homeostasis. However, cells can eliminate excessively stressed mitochondria via autophagosome encapsulating and lysosome degradation, maintaining cell homeostasis [10–12]. In addition, lysosomes can eliminate pathogens encapsulated by autophagosomes [13, 14]. There is increasing evidence that paraepithelial barrier disruption is one of the main reasons autophagy breaches the epithelial barrier [15–17].

Pathogens can disrupt the integrity of epithelial tight junction proteins in different ways, thereby injuring intact cells and promoting pathogenic infection [18, 19]. The main ways for pathogens to destroy the epithelial barrier TJs include recruiting and cleaving the TJs on the cell membrane [20], inhibiting tight junction protein transcription [21, 22], promoting TJs ubiquitination and destruction [23, 24], enhancing mass cytokine production to make TJs redistribute [25]. In addition, recent studies have shown that autophagy can degrade long-lived intracellular proteins such as TJs and increase epithelial barrier permeability [16, 26–30].

Our study aimed to test the hypothesis that *G*. *parasuis* serotype 5 SQ strain (HPS5-SQ) breaking through the porcine respiratory epithelial barrier is linked to autophagy and Claudin-1. Our results indicate that HPS5-SQ could induce mitochondrial oxidative stress, autophagy, and a decrease of Claudin-1 in both porcine lung and swine tracheal epithelial cells (STEC). Autophagosomes could encapsulate and degrade the cytoplasm Claudin-1. A decrease in cytoplasmic Claudin-1 made cell membrane Claudin-1 not be replenished, resulting in damage to the respiratory tract epithelial barrier.

## Results

### HPS5-SQ infection causes damage to the porcine respiratory epithelial barrier

**Fig 1:**
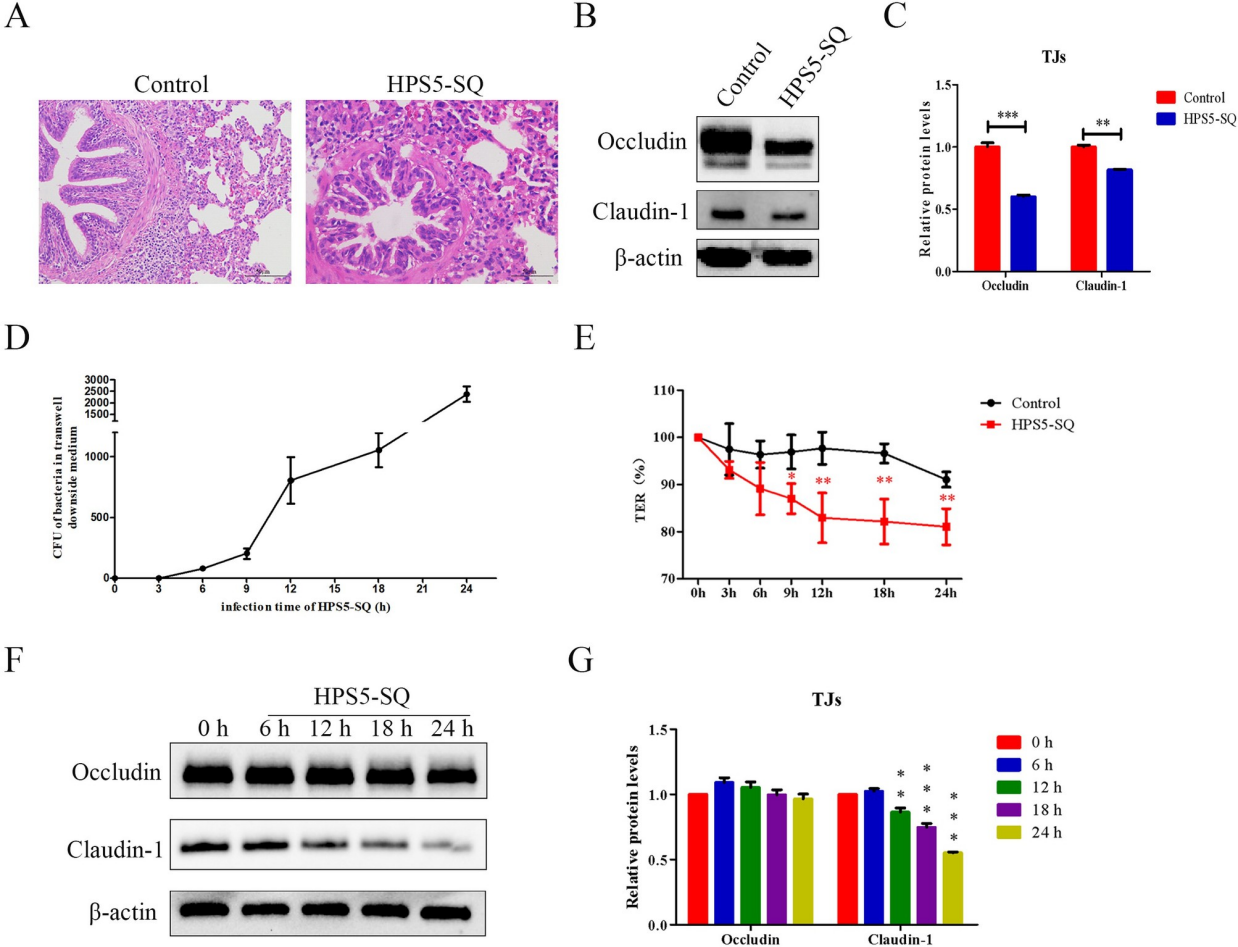
HPS5-SQ infection causes damage to the porcine respiratory epithelial barrier. (A) H&E staining of healthy piglets and piglets infected with HPS5-SQ. Scale bar: 50 μm. (B) Piglets’ lung Occludin and Claudin-1 levels were determined by Western blot. (C) Quantification of Occludin and Claudin-1. Data shown are means ± SEM; ** *P* < 0.01; *** *P* < 0.001. (two-tailed Student’s *t*-tests). (D) CFU of HPS5-SQ across the porcine respiratory epithelial barrier model were counted. Data shown are means ± SEM. (E) The porcine respiratory epithelial barrier was uninfected or infected with HPS5-SQ (MOI of 100). TER of the epithelial barrier was measured. Data shown are means ± SEM; * *P* < 0.05; ** *P* < 0.01. (two-way ANOVA). (F) STEC were either control-treated or infected by HPS5-SQ at an MOI of 100, and whole-cell extracts were prepared from control-infected and HPS5-SQ-infected cells at 6, 12, 18, and 24 hpi. Occludin and Claudin-1 levels were determined by Western blot. (G) Quantification of Occludin and Claudin-1. Data shown are means ± SEM; ** *P* < 0.01; *** *P* < 0.001. (one-way ANOVA).

To determine that HPS5-SQ breached the porcine epithelial barrier and caused porcine lung injury via a paracellular pathway, we firstly assayed piglets’ lung damage and TJs expression by H&E stain and western blot in the infection of HPS5-SQ. HPS5-SQ infection resulted in disordered porcine respiratory epithelial cells, suggesting disruption to the porcine respiratory epithelial barrier, according to H&E staining of swine lung tissue sections (Fig 1A). Western blot results showed significantly decreased porcine lung Occludin and Claudin-1 (Fig 1B and 1C). Then we counted the CFU of HPS5-SQ crossing porcine respiratory epithelial barriers and measured the porcine epithelial barrier’s transepithelial electrical resistance (TER) to confirm that HPS5-SQ infection significantly damaged the porcine respiratory epithelial barrier for 12 h (Fig 1D and 1E). At the same time, STEC Claudin-1 decreased in the infection of HPS5-SQ for 12 h (Fig 1F and 1G).

### HPS5-SQ infection degrades cytoplasm Claudin-1 in STEC

**Fig 2:**
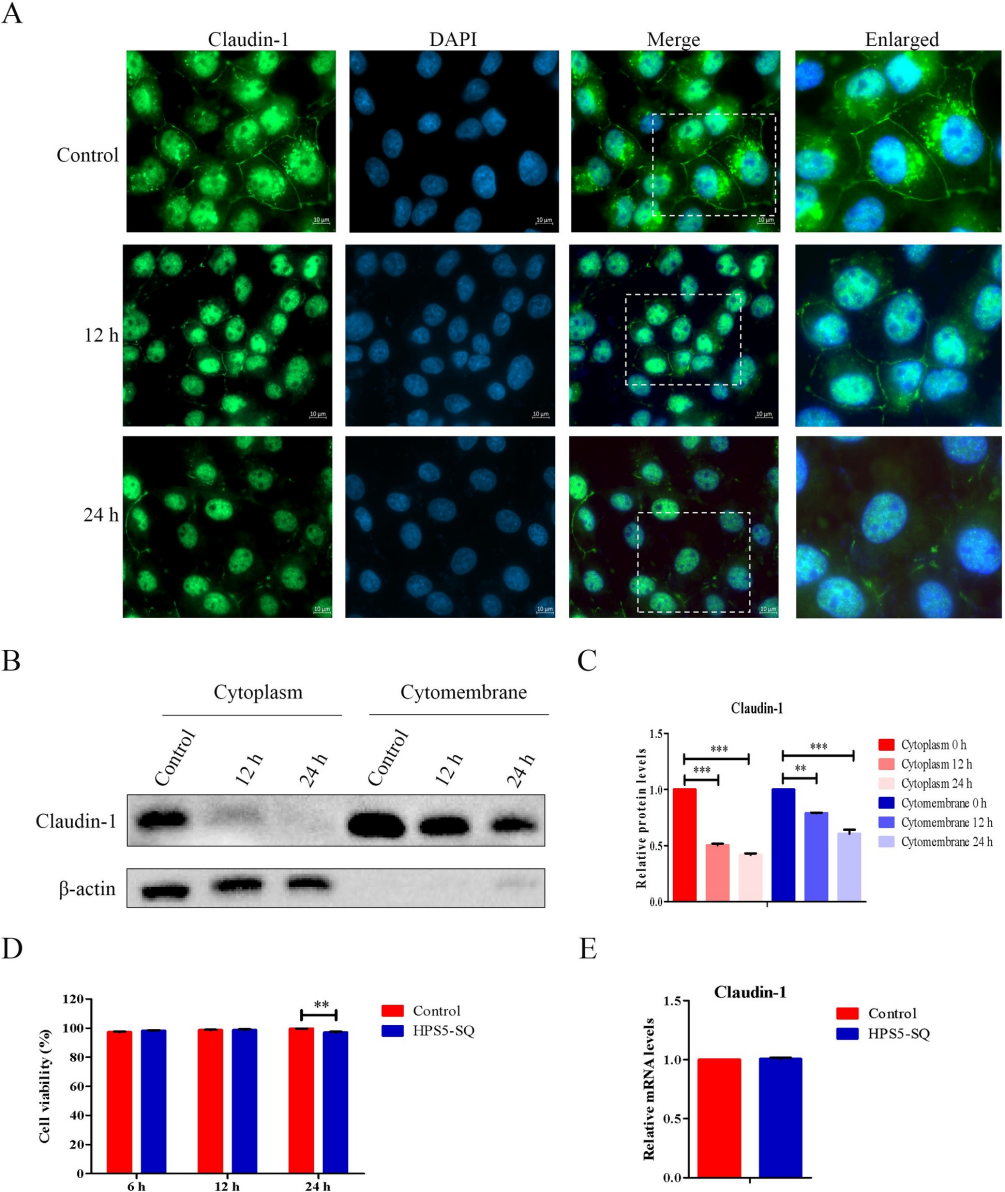
HPS5-SQ infection degrades cytoplasm Claudin-1 in STEC. (A) STEC were infected with control or HPS5-SQ (MOI 100) for 12 h and 24 h in media. STEC were fixed and subjected to immunofluorescence analysis to detect Claudin-1 (green). Scale bar: 10 μm. (B) STEC were control-treated or infected by HPS5-SQ at an MOI of 100, and whole-cell extracts were prepared from control-infected and HPS5-SQ-infected cells at 12 and 24 hpi. (C) Quantification of cytoplasm Claudin-1 and cytomembrane Claudin-1. Data shown are means ± SEM; ** *P* < 0.01; *** *P* < 0.001. (one-way ANOVA). (D) STEC were uninfected or infected with HPS5-SQ (MOI of 100). LDH of the STEC was measured. Data shown are means ± SEM; ** *P* < 0.01. (two-tailed Student’s *t*-tests). (E) STEC were either control treated or infected by HPS5-SQ at an MOI of 100. mRNA levels of Claudin-1 were detected using qRT-PCR.

Indirect immunofluorescence staining (IFA) and Western blot were used to detect Claudin-1 in cells to explore the effect of HPS5-SQ infection on STEC Claudin-1. IFA and Western blot analysis in the porcine tracheal epithelial cells infection model showed a downregulated Claudin-1 level in STEC cytoplasm and cell membrane. In cells treated with *G*. *parasuis* for 12 h or 24 h, the integrity of Claudin-1 in the cell membrane was damaged (Fig 2A–2C). To determine that HPS5-SQ breached the epithelial barrier via a paracellular pathway rather than epithelial cell death in 12 h, we examined the release of lactate dehydrogenase (LDH) into a cell culture medium to evaluate the cytotoxic effects of HPS5-SQ on STEC. The results showed that LDH was not released until 12 h (Fig 2D). The transcription of the *claudin-1* was the same as the uninfected group in 12 h (Fig 2E). The above results indicated that HPS5-SQ infection for 12 h did not down-regulate the protein level of Claudin-1 by inhibiting the STEC *claudin-1* transcription level but rather degrading cytoplasm Claudin-1 in STEC.

### Autophagy cuts off the replenishment of Claudin-1 on the cell membrane

**Fig 3:**
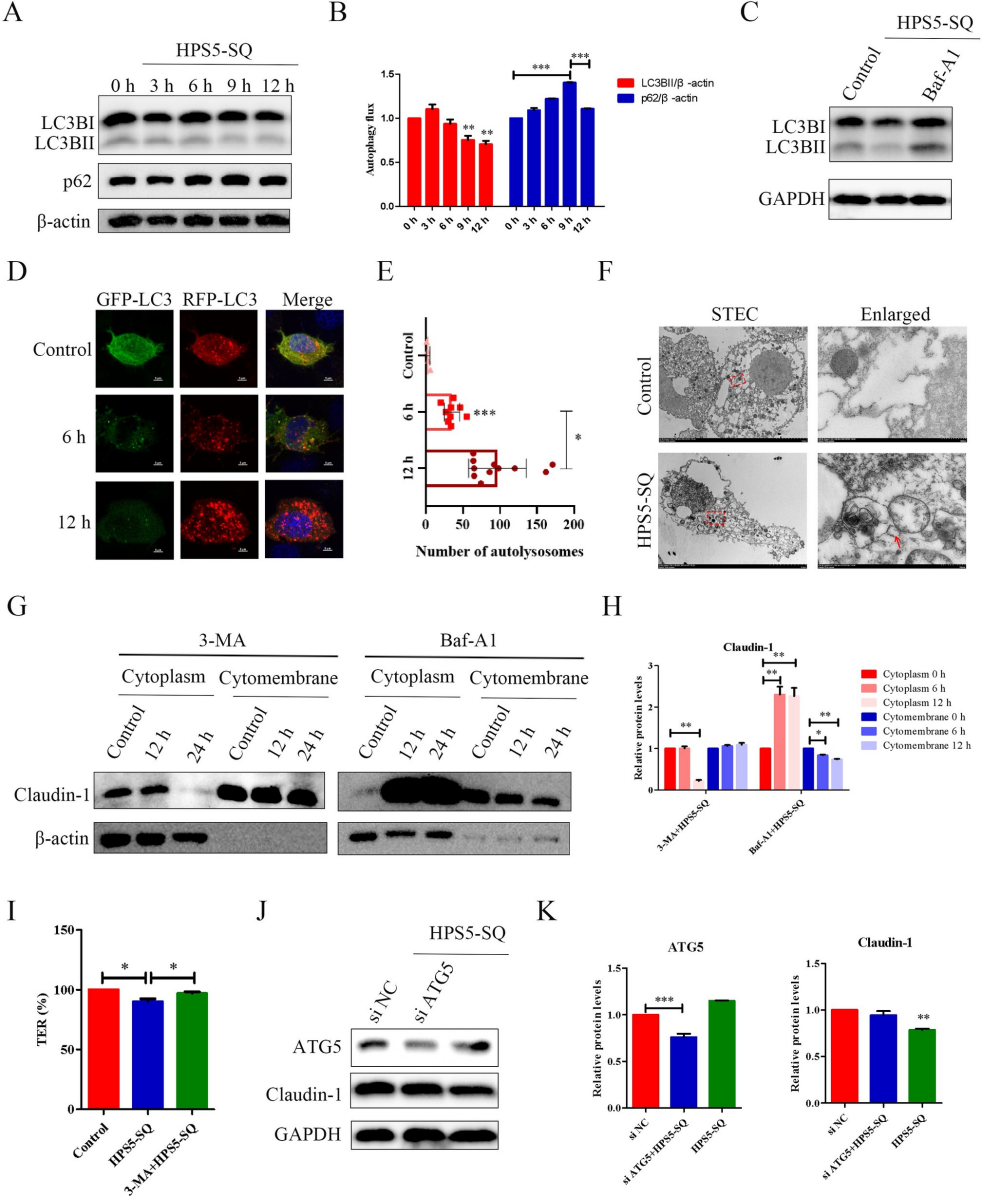
Autophagy cuts off the replenishment of Claudin-1 on the cell membrane. (A) STEC were either controlled or infected by HPS5-SQ at an MOI of 100; whole-cell extracts were prepared from control-infected and HPS5-SQ-infected cells at 3, 6, 9, and 12 hpi. LC3BII and p62 levels were determined by Western blot. (B) Quantification of LC3BII and p62. Data shown are means ± SEM; ** *P* < 0.01; *** *P* < 0.001. (one-way ANOVA). (C) STEC were treated with or without Baf-A1 for the final 4 h and then infected with or without HPS5-SQ (MOI of 100); whole-cell extracts were prepared from control-infected and HPS5-SQ-infected cells at 12 hpi. LC3B levels were determined by Western blot. (D) Immunofluorescence microscopy analysis of autophagosome-like vesicles in STEC infected by HPS5-SQ for 6 and 12 h. Scale bar: 5 μm. (E) Quantification of STEC autolysosomes. Data shown are means ± SEM; * *P* < 0.05; *** *P* < 0.001. (two-tailed Student’s *t*-tests). (F) STEC were either control-treated or infected with HPS5-SQ at an MOI of 100. Electron microscopy images revealed the STEC ultrastructure. In the images, membrane-like vesicles in HPS5-SQ-infected cells were observed. Scale bar: 5 μm, 500 nm. (G) STEC were treated with 3-MA for the final 3 h or Baf-A1 for the final 4 h, then infected with or without HPS5-SQ (MOI of 100); cytoplasm Claudin-1 and cytomembrane Claudin-1 were determined by Western blot. (H) Quantification of cytoplasm Claudin-1 and cytomembrane Claudin-1. Data shown are means ± SEM; * *P* < 0.05; ** *P* < 0.01. (one-way ANOVA). (I) The porcine respiratory epithelial barrier was treated with 3-MA for the final 3 h or untreated with 3-MA, then infected with or without HPS5-SQ (MOI of 100). TER of the epithelial barrier was measured. Data shown are means ± SEM; * *P* < 0.05. (one-way ANOVA). (J) STEC were transfected with ATG5 siRNA or siNC. STEC were infected at MOI = 100. ATG5 and Claudin-1 were determined via Western blot. (K) Quantification of ATG5 and Claudin-1. Data shown are means ± SEM; ** *P* < 0.01; *** *P* < 0.001. (one-way ANOVA).

Recent studies have shown that autophagy impaired barrier function caused by covering and degrading TJs [17, 28]. Here, we explored the mechanisms of autophagy influencing the replenishment of Claudin-1 on the cell membrane. The STEC infected with HPS5-SQ for 12 h had decreased the expression of LC3BII in 12 h. At the same time, the expression of p62 decreased from 9–12 h (Fig 3A and 3B), and Baf-A1 inhibited the decrease of LC3BII in the infection of HPS5-SQ in 12 h (Fig 3C). The above results showed that HPS5-SQ induced STEC complete autophagy and HPS5-SQ infection destroyed the integrity of Claudin-1 with concomitant autolysosome maturation in STEC, which was consistent with an increased number of autolysosomes in the infection of HPS5-SQ in 12 h (Fig 3D–3F). When STEC were infected with HPS5-SQ for 12 h, pretreatment of 3-MA inhibited the degradation of Claudin-1 in both cell membrane and cytoplasm. However, pretreatment with Baf-A1 only prevented the degradation of Claudin-1 in the cytoplasm, and Claudin-1 in the cytomembrane was still decreased in 12 h (Fig 3G and 3H). Cell damage may result in the release of intracellular LDH and Claudin-1 after 24 h infection with HPS5-SQ. Furthermore, it is 3-MA that could inhibit TER decreasing (Fig 3I). When STEC were infected with HPS5-SQ for 12 h, *ATG5* knockdown effectively kept Claudin-1 level (Fig 3J and 3K). These results showed that in the infection of HPS5-SQ, STEC Claudin-1 in the cytoplasm first decreased, and intercellular Claudin-1 was partially damaged, indicating that cytoplasmic Claudin-1 was enveloped and degraded by autophagic degradation and autophagy degrading cytoplasmic Claudin-1 may affect the supply of Claudin-1 to the cell membrane.

### HPS5-SQ-mediated autophagy degrades Claudin-1 covering mitochondria in STEC

**Fig 4:**
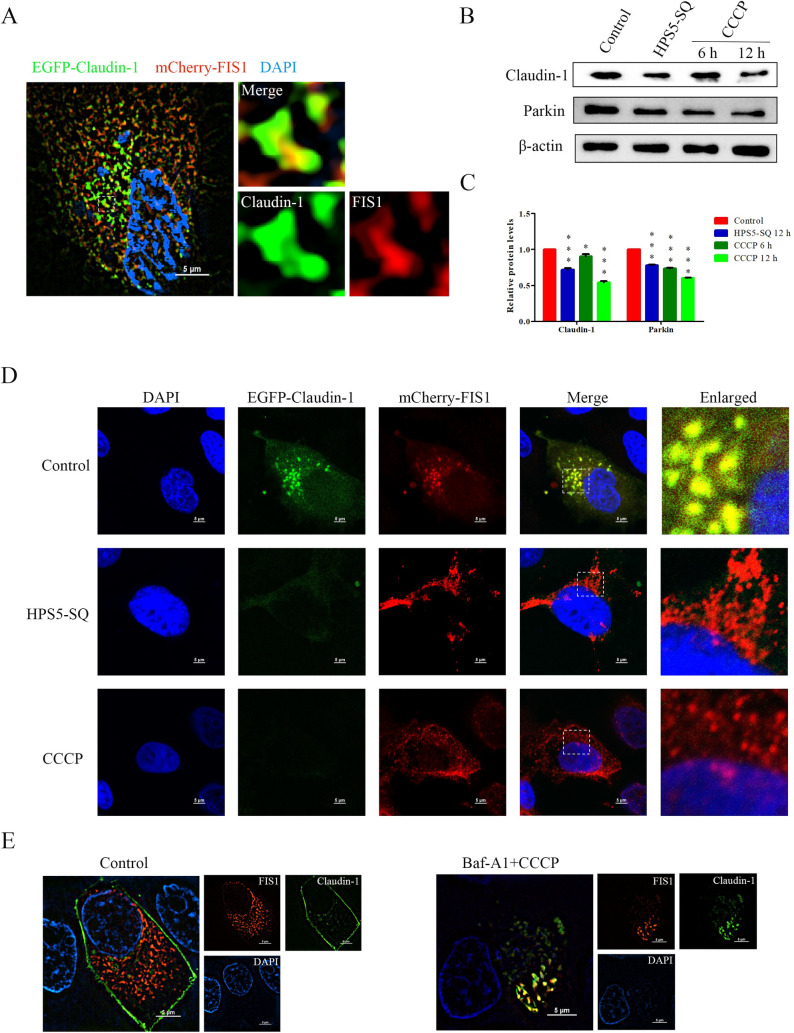
HPS5-SQ-mediated autophagy degrades Claudin-1 covering mitochondria in STEC. (A) The expression of the mCherry-FIS1 and EGFP-Claudin-1 in STEC. Immunofluorescence microscopy images detected the subcellular localization of mitochondria and Claudin-1. Scale bars: 5 μm. (B) Western blot showing Claudin-1 and Parkin expression in HPS5-SQ-infected cells or treated with mitophagy activator: CCCP for 6 and 12 h. (C) Quantification of Claudin-1 and Parkin. Data shown are means ± SEM; * P < 0.05; *** *P* < 0.001. (one-way ANOVA). (D) STEC expressing mCherry-FIS1 and EGFP-Claudin-1 were control-treated or infected with HPS5-SQ (MOI of 100) and treated with CCCP for 12 h as a positive control. Immunofluorescence microscopy images detected the Claudin-1 and mitochondria. Scale bar: 5 μm. (E) The expression of the mCherry-FIS1 and EGFP-Claudin-1 in STEC were treated with Baf-A1 for the final 4 h or untreated with Baf-A1, then CCCP was added to STEC pretreated with Baf-A1 for 12 h. Immunofluorescence microscopy images detected the subcellular localization of mitochondria and Claudin-1 in STEC pretreated with Triton-100 for more than 12 h. Scale bars: 5 μm.

It has been reported that cell protein gathering in the out membrane of mitochondria can be enveloped and degraded with mitophagy [31]. EGFP-Claudin-1 expression showed that STEC Claudin-1 wrapped oval cell structures (S1 Fig). Immunofluorescence analysis of mitochondria and cytoplasm Claudin-1 and expression of mCherry-FIS1 and EGFP-Claudin-1 in STEC demonstrated the co-localization of Claudin-1 covered mitochondrial outer membrane (Figs 4A and S2 and S1 Video). The above results showed that the STEC cellular structure wrapped by cytoplasmic Claudin-1 was mitochondria. In the infection of HPS5-SQ or pretreated with CCCP for 6 and 12 h, Claudin-1 and Parkin level in STEC was downregulated, and EGFP-Claudin-1 expression was quickly diminished (Fig 4B–4D). These results indicated that autophagy induced by HPS5-SQ infection involved cytoplasmic Claudin-1 and mitochondria degradation. Next, we fixed STEC expressing EGFP-Claudin-1 and mCherry-FIS1, which was permeabilized for more than 12 h, the structure of EGFP-Claudin-1 embedded on the cell membrane surface was in a diffuse state. While pretreated with Baf-A1, STEC autophagosomes encapsulated mitochondria and Claudin-1, and the subcellular co-localization of mitochondria and Claudin-1 still existed (Fig 4E). This result indicated that Claudin-1 aggregating on the mitochondrial surface could be degraded by autophagy. Undegraded cytoplasmic Claudin-1 would escape outside the cell after the permeability of the cell membrane increased, which also reduced the supply of Claudin-1 to the cell membrane.

### HPS5-SQ-mediated autophagy envelops mitochondria under oxidative stress and kills invading bacteria

**Fig 5:**
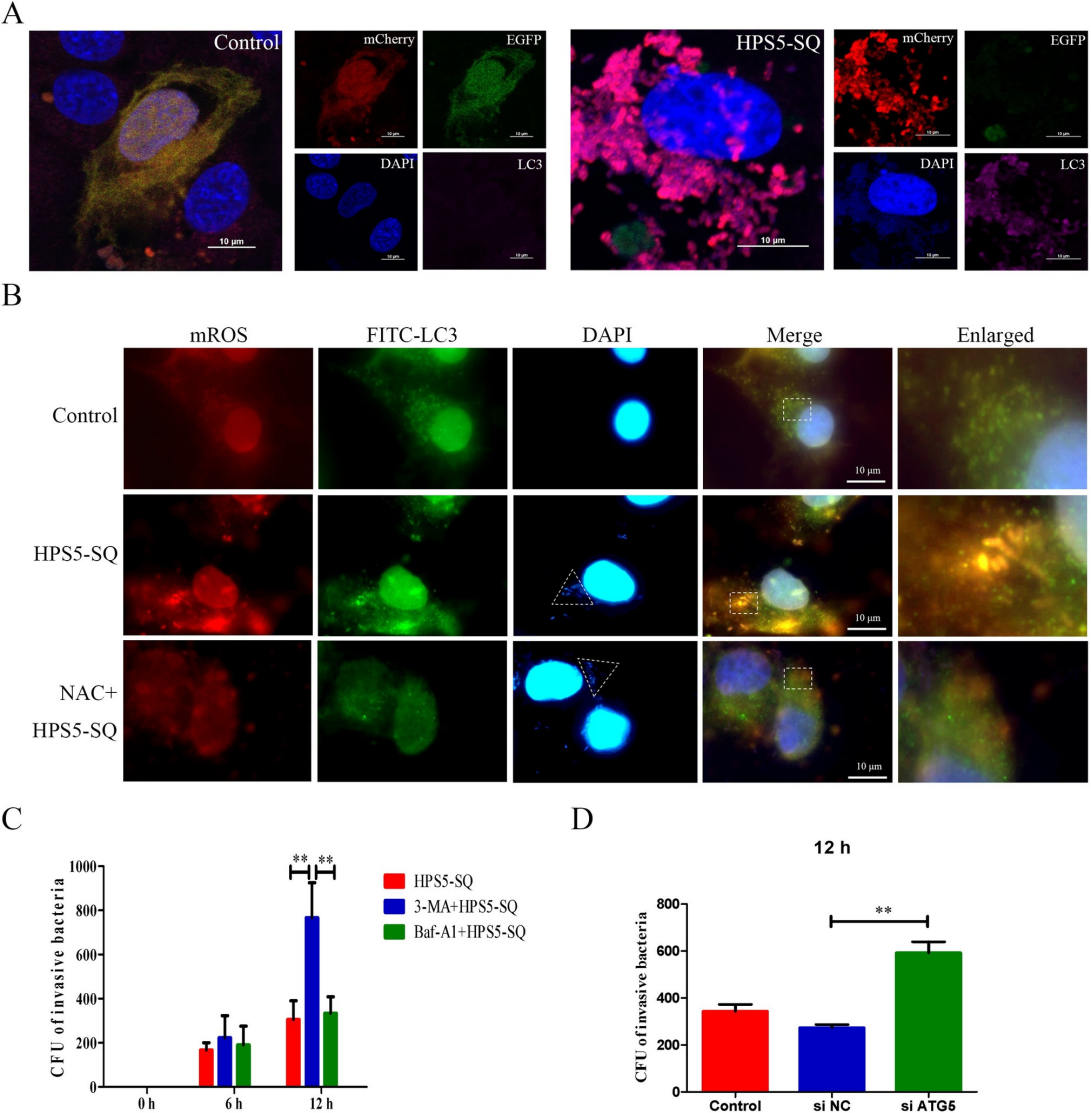
HPS5-SQ-mediated autophagy envelops mitochondria under oxidative stress and kills invading bacteria. (A) STEC were fixed and subjected to immunofluorescence analysis to detect LC3 (pink) and mitochondrial fluorescence (red and green). Scale bar: 10 μm. (B) Immunofluorescence of control, HPS5-SQ, and HPS5-SQ+NAC (5 mM for the final 1 h) infected STEC stained for mROS and LC3B. The white triangle refers to HPS5-SQ (blue). Scale bar: 10 μm. (C) STEC were treated with 3-MA for the final 3 h or Baf-A1 for the final 4 h, quantifying the invading HPS5-SQ in the infection of HPS5-SQ for 6 and 12 h. Data shown are means ± SEM; ** *P* < 0.01. (two-way ANOVA). (D) STEC were treated with ATG5 siRNA for 24 h and infected with HPS5-SQ for 12 h. Quantification of the invading HPS5-SQ. Data shown are means ± SEM; ** *P* < 0.01. (one-way ANOVA).

As shown in Fig 5A, in the infection of HPS5-SQ, fragmented mitochondria were encapsulated by LC3. Then, enhanced green fluorescent protein (EGFP) was diminished, suggesting that autophagy eliminated fragmented mitochondria. IFA results showed that HPS5-SQ (inside the triangle) and oxidative stress mitochondria were encapsulated by LC3. However, NAC reversed this effect (Fig 5B). The autophagosome’s formation and degradation were inhibited to explore the underlying mechanism of autophagy-induced bacteria killing. Pretreatment with Baf-A1 did not affect the number of invading HPS5-SQ. Nevertheless, pretreatment of 3-MA had more invading HPS5-SQ, suggesting that bacteria were killed in autophagosomes and mitochondria under oxidative stress. Releasing mROS may help kill invading HPS5-SQ (Fig 5C). When STEC were infected with HPS5-SQ for 12 h, *ATG5* knockdown effectively increased the invading HPS5-SQ (Fig 5D). The results indicated that HPS5-SQ-mediated autophagy enveloped mitochondria under oxidative stress and killed invading bacteria.

### HPS5-SQ-mediated autophagy involves the decline of porcine respiratory epithelial Claudin-1

**Fig 6:**
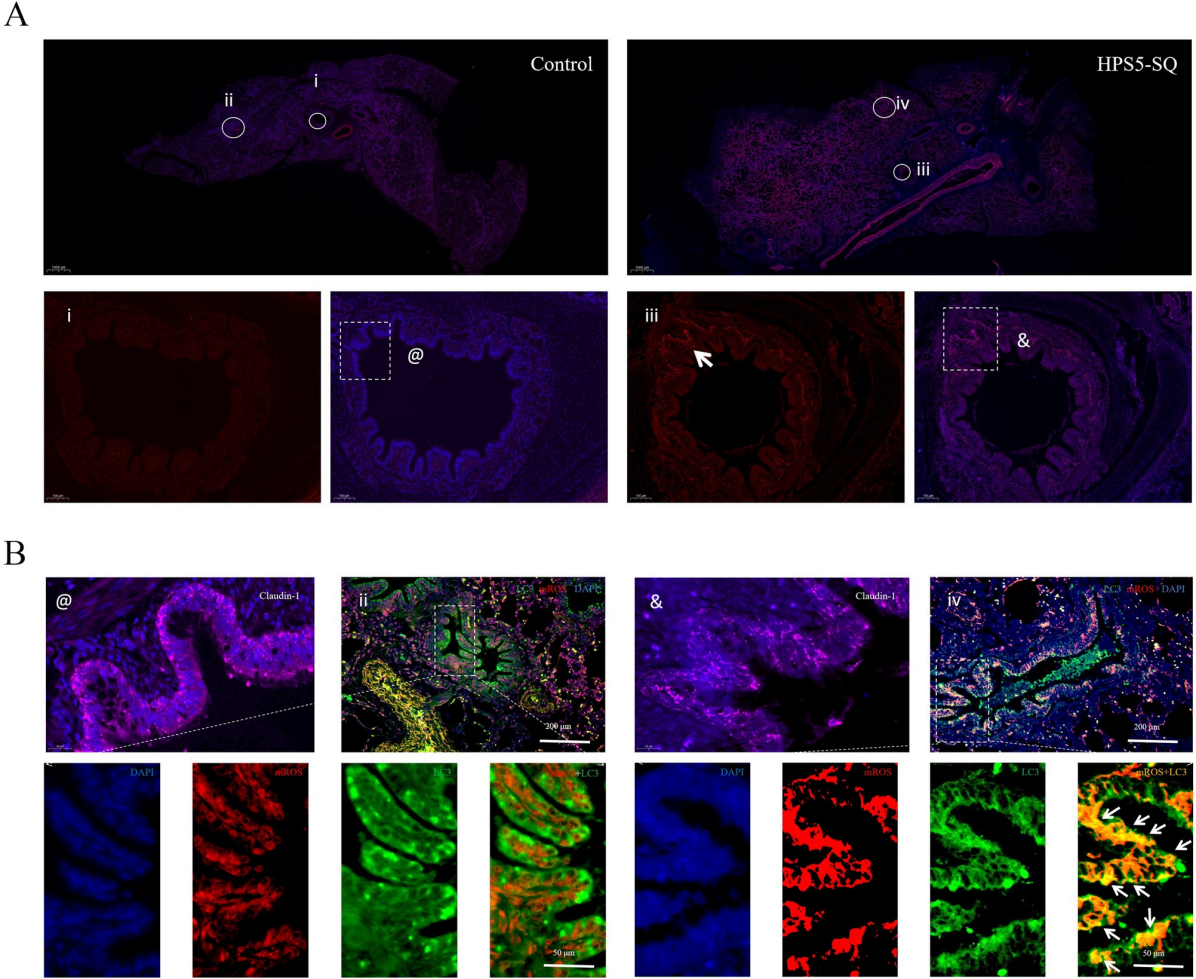
HPS5-SQ-mediated autophagy involves the decline of porcine respiratory epithelial Claudin-1. (A) The mROS staining of piglets’ lung tissue section of the autopsy showed the mROS (red) and the nucleus (blue). Scale bars: 1000 μm, 100 μm. (B) Pictures were marked by @ and & in Fig 6A, and immunofluorescence microscopy detected the Claudin-1 (pink) and DAPI (blue) in piglets lung tissue. The picture was marked by ii and iv in Fig 6A, and immunofluorescence microscopy detected the localization of mROS(red), LC3(green), and DAPI (blue) in healthy piglets lung tissue infected with HPS5-SQ. Scale bars: 200 μm, 50 μm, 20 μm.

In the infected group, piglets infected with HPS5-SQ and mROS were generated in porcine lung tissue, suggesting mitochondrial oxidative stress in piglets’ lungs (Fig 6A). IFA staining of lung tissue sections indicated that Claudin-1 was severely damaged with mitochondrial oxidative stress. Importantly, the oxidatively stressed mitochondria co-localized with LC3 (Fig 6B). These results revealed a causal link between oxidative stress and Claudin-1 damage, where autophagy may be activated by mROS and involved in the decline of mROS and Claudin-1.

## Discussion

As the high-virulence *G*. *parasuis* serotype, HPS5-SQ is more likely to penetrate the respiratory epithelial barrier and produce systemic infection, especially lung infection [2]. Therefore, it is important to investigate the underlying mechanism of *G*. *parasuis* breaking through the respiratory barrier. Our study found that HPS5-SQ infection can cause reduced levels of TJs, impair TJs integrity in piglets’ lungs, and break through the epithelial barrier of piglets’ lungs. During early HPS5-SQ infection, autophagy cutting off cell membrane Claudin-1 replenishment disrupted STEC cytoplasmic Claudin-1 and breached the porcine airway epithelial barrier by injuring paracellular.

Previous studies have shown ROS aggregation after bacteria infection [32, 33]. Mitochondria-derived antimicrobial effectors like mROS primarily generated during mitochondrial metabolism aid in bacterial killing [34]. When *G*. *parasuis* infected, released ROS gathered at the bacterial site to resist the bacteria [35]. However, the aggregation of mitochondria oxidative catastrophe induces cell death and inflammation [36]. Cellular mROS can trigger autophagy to remove damaged organelles and excess mROS [37]. Inhibition of ROS production may reduce autophagy [38]. During autophagy in epithelial cells, mitochondria and mROS are confined in autophagosomes and then degraded by lysosomes to maintain cell homeostasis [10, 12, 36, 39]. mROS-resisting bacteria invasion is eliminated by autophagy, resulting in decreased mROS, which could explain why the invasion of *G*. *parasuis* is time-dependent [12, 40]. According to our findings, HPS5-SQ-infected STEC activated mitochondrial oxidative stress, and epithelial cells removed oxidative stress mitochondria and mROS by autophagy, consistent with Kraft’s study [12]. Therefore, the invasion of *G*. *parasuis* was related to the virulence and invasive ability of the bacteria and mROS in epithelial cells.

Autophagy regulates TJs’ expression and the epithelial barrier’s function [41]. In the process of pathogens infection, epithelial cell mitochondria disruption causes damage to the epithelial barrier [42]. Our study discovered that autophagy degraded cytoplasmic Claudin-1, prevented Claudin-1 replenishment in the cell membrane, and damaged the respiratory tract epithelial barrier [8, 43]. When *G*. *parasuis* infected STEC for 12 h, autolysosomes destroyed Claudin-1 in the cytoplasm, decreasing the Claudin-1 in STEC. 3-MA has been previously confirmed to inhibit STEC autophagy and cell membrane Claudin-1 degradation. Autophagy contents are sequestered by double-membrane compartments during autophagosome formation and cannot function as normal cellular structures [17]. Autophagic degradation of Claudin-1 may block its recycling back to the cytoplasm.

The distribution of Claudin-1 in cells is closely related to the calcium ion concentration [44]. Extracellular calcium ions pull Claudin-1 into the cytoplasm to form tight junctions on the cell membrane [44–46]. In cells, the calcium ion concentration in the mitochondrial is second to the endoplasmic reticulum, and the calcium ion concentration in the nucleus is higher than cytoplasmic. Calbindin is sub-localized in the outer mitochondrial membrane by calcium ions in the mitochondria; hence calcium ions in the mitochondria may also cause Claudin-1 sub-localization in the outer mitochondrial membrane [44, 47]. The concentration of calcium ions in extracellular is higher than cytoplasmic, which may be the reason for cell damage accelerating the escape of cytoplasmic Claudin-1 and the encapsulation of autophagy-related proteins inhibiting the escape of cytoplasmic Claudin-1. Our data found that Claudin-1 was wrapped in the outer mitochondrial membrane by observing the co-localization of STEC expressing mCherry-FIS1 and EGFP-Claudin-1. When the permeability of the cell membrane increases, extracellular calcium ions may pull cytoplasm Claudin-1, and the structure of Claudin-1 wrapped around mitochondria could not be maintained for too long.

Parkin-dependent mitophagy is induced by the PINK1-Parkin signaling pathway [48]. Parkin is ubiquitinated and recruited from the cytoplasm to the outer mitochondrial membrane during mitophagy. On the mitochondrial outer membrane, ubiquitinated Parkin binds to p62, and both of them are wrapped by LC3. Ultimately, Parkin and mitochondria are degraded by autolysosomes [49]. Our study found that, widely, HPS5-SQ infection can lead to quenching of mCherry-EGFP-FIS1 green fluorescence. Therefore, HPS5-SQ infection is more likely to activate mitophagy, not autophagy, to degrade mitochondria. Inhibition of mitophagy by knocking out *ATG5* or *parkin* increased the number of invading bacteria [50]. HPS5-SQ was killed in autophagosomes without lysosomal degradation, indicating that cells kill invading pathogens by confining mitochondria and pathogens.

**Fig 7:**
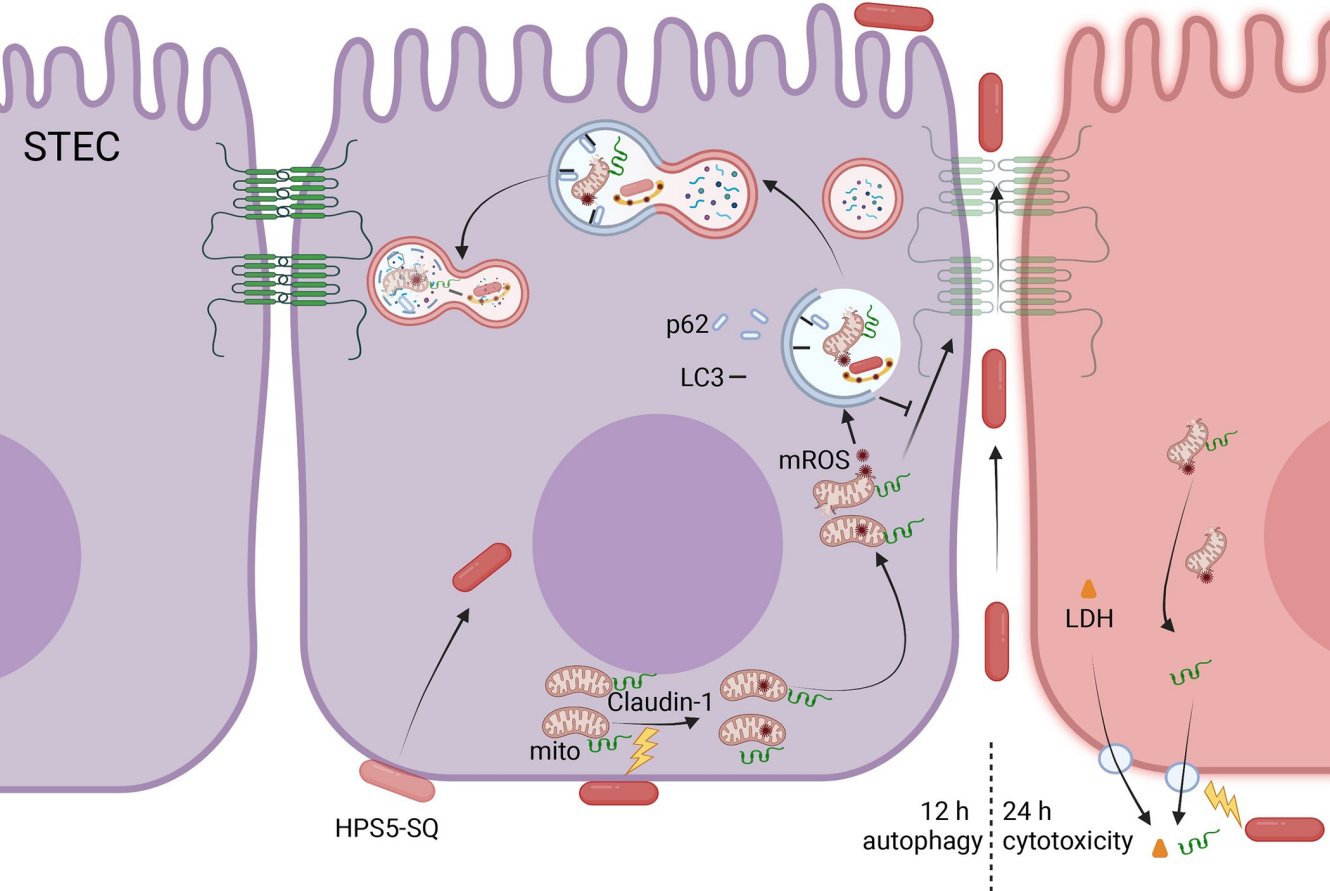
Diagram of the destruction of the respiratory epithelial barrier in the infection of HPS5-SQ. Autophagy induced by mROS facilitates the damage of the respiratory epithelial barrier in the infection of HPS5-SQ. In brief, autolysosomes can degrade mitochondrion and Claudin-1. In addition, cytotoxicity induced by HPS5-SQ infection accelerates the escape of Claudin-1 from the cytoplasm. Consequently, the Claudin-1 degradation and escape in the STEC make cell membrane Claudin-1 unable to replenish in time, and the respiratory tract epithelial barrier is damaged.

Our research proposed a model that *G*. *parasuis* infection led to a breach in the porcine respiratory epithelial barrier. Autolysosomes of STEC blocked the replenishment of membrane Claudin-1 from the cytoplasm (Fig 7). Next, we will deplete calcium within the mitochondria and close the mitochondrial surface calcium channels to explore whether the accumulation of Claudin-1 around mitochondria is related to the traction effect of a high concentration of calcium ions. In addition, the process that mitophagy signaling pathway protein Pink1 aggregates in the mitochondrial outer membrane and is degraded by autophagy will be tested in our following experiments.

## Materials and methods

### Ethics statement

All animal experiments have been approved by the Animal Experiment Ethics Committee of Nanjing Agricultural University under the China Animal Welfare Committee guidelines. The animal ethics audit number is PTA2020038.

### Cells and bacterial strains

Swine tracheal epithelial cells (STEC; affandi-e, x1204502), purchased from affandi-e, were cultured in Dulbecco’s modified Eagle’s medium (DMEM; Gibco, C11995500BT) plus 10% fetal bovine serum (FBS; Gibco, 10270106) at 37°C under 5% CO_2_. The HPS5-SQ strain was isolated from a sick pig in Jiangsu (Suqian, China) and stored in our laboratory (Nanjing Agricultural University, Nanjing, China), which is grown in TRYPTONE SOYA BROTH (TSB; OXOID, CM0129) containing 0.1 mg/mL Nicotinamide adenine dinucleotide (NAD; Beyotime, ST1110) with 5% fetal bovine serum (FBS; PAN biotech, P30-3302).

### Chemical reagents and antibodies

Opti-MEM (31985088) was purchased from Gibco. MitoTracker Red CMXRos (40740ES50) was purchased from YEASEN. DAPI (C1002) and N-acetylcysteine (S0077) were purchased from Beyotime. 3-methyladenine (3-MA) (S2767) and bafilomycin A1 (Baf-A1) (S1413) were purchased from Selleck Chemicals. CCCP (C2759) (SAB5701328) was purchased from Sigma-Aldrich. Anti-Occludin (91131S), Claudin-1 (13255S), GAPDH (5174S), Parkin(2132S), and β-actin (3700S) were purchased from Cell Signaling Technology. Anti-P62 (T55546), ATG5 (T55766), and LC3B primary antibodies were purchased from Abmart.

### Animal challenge experiments

Eight healthy weaned Large White × Landrace × Duroc hybrid piglets aged 14–21 days were purchased from Nantong, tested before use by *G*. *parasuis* detection kit (Biovet Ltd., China), *Streptococcus suis* type 2 detection kit (Biovet Ltd., China), PCV2 detection kit (Keqian Ltd., China), PRRSV detection kit (Keqian Ltd., China). All piglets were seronegative for *G*. *parasuis*, *Streptococcus suis* type 2, PCV2, and PRRSV. All piglets were randomly divided into the HPS5-SQ infection group (n = 5) and the control group (n = 3). HPS5-SQ was intraperitoneally inoculated one week later, and the total dose was 4×10^9^ CFU [2]. An autopsy was done for infected dead piglets, and euthanized the surviving piglets 14 days after infection. Lung tissue was collected for histopathological examination, and the indirect immunofluorescence staining of pig lung tissue was done by Wuhan Bolfu Biotech Co., Ltd.

### Cell treatment

STEC were cultured as a monolayer on a 12-well plate (Corning, I0905-191). For infection, cells were first washed with serum-free DMEM and incubated with bacteria in serum-free DMEM at 37°C. Cell death was examined by measuring lactate dehydrogenase release using a Cytotoxicity Detection kit (Beyotime, C0017). For autophagy analysis, a medium containing 10 μM CCCP for 6 and 12 h, 5 mM NAC for 1 h, 3 mM 3-MA for 3 h, or 1 μM Baf-A1 for 4 h.

### Construction of a porcine respiratory epithelial barrier model

The STEC were digested from the cell flask and then diluted. 500 μL of the diluted solution was seeded in the upper chamber of the Transwell chamber (Corning, 3401). 1.5 mL of DMEM medium containing 10% FBS was added to the lower chamber, and the cells were cultured at 37°C under 5% CO_2_. In the early stage of barrier construction, the medium is changed every 2 days, and the cell resistance meter (Millicell-ERS, MERS00002) is used to detect the barrier resistance value. When the resistance value increases, the medium needs to be replaced daily to provide nutrients, and the barrier resistance value is measured daily. The corresponding drug pretreatment and experimental infection occur when the value is stable.

### HPS5-SQ translocation assay

Experiments were performed when the porcine respiratory epithelial barrier was constructed. Transwells containing STEC were infected with HPS5-SQ at an MOI of 100 for 3, 6, 9, 12, 18, and 24 h. CFU of HPS5-SQ crossing porcine respiratory epithelial barriers to the basolateral chamber of the transwells was counted on supplemented TSA, and the porcine epithelial barrier’s transepithelial electrical resistance (TER) was measured. Assays were performed in triplicate and repeated three times.

### Immunofluorescence staining and probe staining

STEC were cultured as a monolayer on a confocal dish (NEST, 801002) and pretreated with drugs or infected with HPS5-SQ. After the infection, the culture medium was discarded. The cells were washed three times with pre-cooled PBS, fixed with 4% paraformaldehyde for 15 min, then blocked with 5% skimmed milk (Difco, 232100) in a 37°C incubator for 1 h. The blocked samples were incubated with the primary antibody (1: 100) overnight at 4°C. The next day, the unbound primary antibody was washed with PBST. The cells were incubated with Alexa Flour 488 labeled mouse antibody (1: 200; Beyotime, A0428), Cy3 labeled rabbit antibody (1:200; Beyotime, A0516), Alexa Flour 647 labeled rabbit antibody as secondary antibodies (1:200; Beyotime, A0468), at room temperature for 60 min. Unbound secondary antibodies were washed with PBST, and mitochondria with reactive oxygen species were stained with MitoTracker Red CMXRos(500 nM). DAPI (1: 1000) was used for nuclear staining. The STEC samples were observed and photographed under an immunofluorescence microscope (Leica, Solms, Germany), and the images were analyzed using Image J.

### The fusion expression of cellular proteins with fluorescent proteins

FIS1 is a bonafide endpoint mitophagy reporter in cultured cells and tissues. Full-length cDNA encoding FIS1 was amplified by RT-PCR from the total RNA of STEC and cloned into pCMV-N-mCherry and pCMV-N-mCherry-EGFP. Full-length cDNA encoding Claudin-1 was amplified by RT-PCR from the total RNA of STEC and cloned into pEGFP-C3. The 2 empty vectors (pEGFP-C3 [YouBia, VT1109] and pCMV-N-mCherry [Beyotime, D2711]) were stored in our laboratory. pCMV-N-mCherry-EGFP was constructed using PCR-based homologous recombination methods. Primers used for the plasmid construction experiment are listed in the S1 Table. Lentivirus LV-mRFP-GFP-LC3 (pig) is coated by Hanbio [51]. STEC were infected by Lentivirus LV-mRFP-GFP-LC3 (MOI 1). mCherry-FIS1 (1 μg), EGFP-Claudin-1 (1 μg), and mCherry-EGFP-FIS1 (1 μg) plasmid were transfected into STEC with Lipo2 000 (Invitrogen, 11668019). Then the corresponding pretreatment and infection tests were carried out 12 h after the lentivirus infection or transfection. The STEC samples were observed and photographed under an immunofluorescence microscope (Olympus, Tokyo, Japan). The images were analyzed using Image J, and the numbers of autophagosomes and autolysosomes were analyzed using Graph Pad Prism5 (Graph Pad, San Diego, CA).

### Transmission electron microscope (TEM)

To detect the effect of HPS5-SQ on STEC structure, STEC were cultured in a 75 cm^2^ cell culture flask. Briefly, uninfected or infected HPS5-SQ STEC were scraped, embedded in low-temperature agarose (Sigma, A9414), and fixed with 2.5% glutaraldehyde diluted in PBS. Cell pellets were dehydrated. Then, ultrathin-slices were cut and observed under a JEM-1400 transmission electron microscope (JEM, Tokyo, Japan).

### Invasion assays

STEC were distributed into 24-well plates and incubated at 37°C in 5% CO_2_ until the cells were confluent. Cells were then inoculated with HPS5-SQ at MOI of 100. After 6 and 12 h of incubation at 37°C with 5% CO_2_, cells were washed three times with PBS to remove nonadherent bacteria. Extracellular bacteria were killed by adding DMEM containing gentamicin (100 μg/mL; Sigma, G3632) and penicillin G (10 μg/mL; Sigma, A9518) for an additional 1 h. To determine bacterial counts, antibiotic-treated cells were washed three times in PBS, lysed with double-distilled water, and diluted appropriately with PBS before spreading on supplemented TSA. Assays were performed in triplicate and repeated three times.

### siRNA transfection

siATG5 (sense, 5′-CCCUCUAUCAGGAUGAGAUTT-3′; antisense, 5′-AUCUCAUCCUGAUAGAGGGTT-3′) synthesized from Shanghai Hanbio was used to knock down the expression of *ATG5* (5′-CCCTCTATCAGGATGAGAT-3′) in porcine cells and inhibit autophagy [52]. siATG5 was added to STEC after 15 min incubation with Lipo2000, and STEC was washed 3 times with PBS and transfected with 50 nM (final concentration) siATG5 for 24 h, followed by HPS5-SQ infection. Negative Control siRNA (sense, 5′-UUCUCCGAACGUGUCACGUTT -3′; antisense, 5′-ACGUGACACGUUCGGAGAATT-3′) from Shanghai Hanbio was used as the control.

### SDS-PAGE and Western blot

The medium in the cell sample was discarded, the STEC were washed twice with pre-cooled PBS, lysed with PMSF (1 mM; Beyotime, ST506) cell lysate RIPA (Beyotime, P0013) for 10 min on ice, and the cell protein concentration was measured with BCA (Beyotime, P0006). 5×SDS-PAGE loading buffer was added to the cell lysate and incubated in a water bath at 100°C for 10 min, and 10 μg protein was loaded per well for SDS-PAGE and then transferred to PVDF (Millipore, ISEQ00010) membrane by a semi-dry transfer machine (Bio-Rad, 1703957). The PVDF membrane was blocked with 5% skim milk, then incubated with the primary antibody at 4°C overnight. The unbound primary antibody (1: 2000) was washed off with PBST. The membrane was incubated with the secondary antibody at room temperature for 2 h. The unbound secondary antibody (1: 5000) was washed off with PBST. Finally, ECL chemiluminescence solution (Beyotime, P0018A) was added to the PVDF membrane for exposure (Bio-Rad, 1708280), and bands were analyzed with Image J for quantitative analysis.

### RNA isolation and RT-qPCR analysis

We extracted STEC total RNA with Total RNA Extraction Reagent (Vazyme, R401) and got STEC cDNA with HiScript II Q RT SuperMix Kit (R222-01). Real-time quantitative PCR reactions were performed using hamQ Universal SYBR qPCR Master Mix Kit (Vazyme, Q711-00) and a StepOne real-time PCR system (Applied Biosystems, ABI StepOne). All measurements were duplicated and calculated using the arithmetic mean of Ct values. As an experimental control, the target gene Ct value was divided by the corresponding internal reference gene (*GAPDH*) Ct value. Using a relatively quantitative method, the obtained values were exponentiated 2^-ΔΔCt^ compared to the control 2^-ΔΔCt^. The assay was performed in biological triplicate, and error bars represent SEM. Primers used for PCR are listed in the S2 Table.

### Statistical analysis

All experiments were performed three times independently. Data were expressed as mean ± SEM, and GraphPad Prism 5 was used for one-way analysis of variance (ANOVA), two-way ANOVA, and two-tailed Student’s *t*-tests. *P*-value < 0.05 is considered statistically significant, *P*-value < 0.01 is highly significant, *P*-value < 0.001 is extremely significant.

## Supporting information

S1 Fig The cellular distribution of Claudin-1 in the layer-by-layer scanning. STEC were transfected with indicated EGFP-Claudin-1 for 12 h. STEC were fixed and subjected to immunofluorescence analysis to detect Claudin-1 (green). Scale bar: 10 μm.
(TIF)

S2 Fig The co-localization of STEC mitochondria and Claudin-1. STEC infected with HPS5-SQ for 12 h were fixed and subjected to immunofluorescence analysis to detect Claudin-1 (green) and mitochondria (red) by staining Claudin-1 and mROS. Scale bar: 10 μm.
(TIF)

S1 Video The scan reconstruction video for STEC. STEC were transfected with the mCherry-FIS1 and EGFP-Claudin-1 for 12 h. Immunofluorescence microscopy images detected the cellular localization of mitochondria and Claudin-1.
(MP4)

S1 Table The sequence of primers for the plasmid construction experiment. (DOCX)

S2 Table The sequence of primers for quantitative RT-PCR. (DOCX)
