# Supplementary figures and images for: Glaesserella parasuis serotype 5 breaches the porcine respiratory epithelial barrier by inducing autophagy and blocking the cell membrane Claudin-1 replenishment
Source: PLoS Pathog. 2022 Oct 13;18(10):e1010912. doi: 10.1371/journal.ppat.1010912 (PMC9595547; doi:10.1371/journal.ppat.1010912)

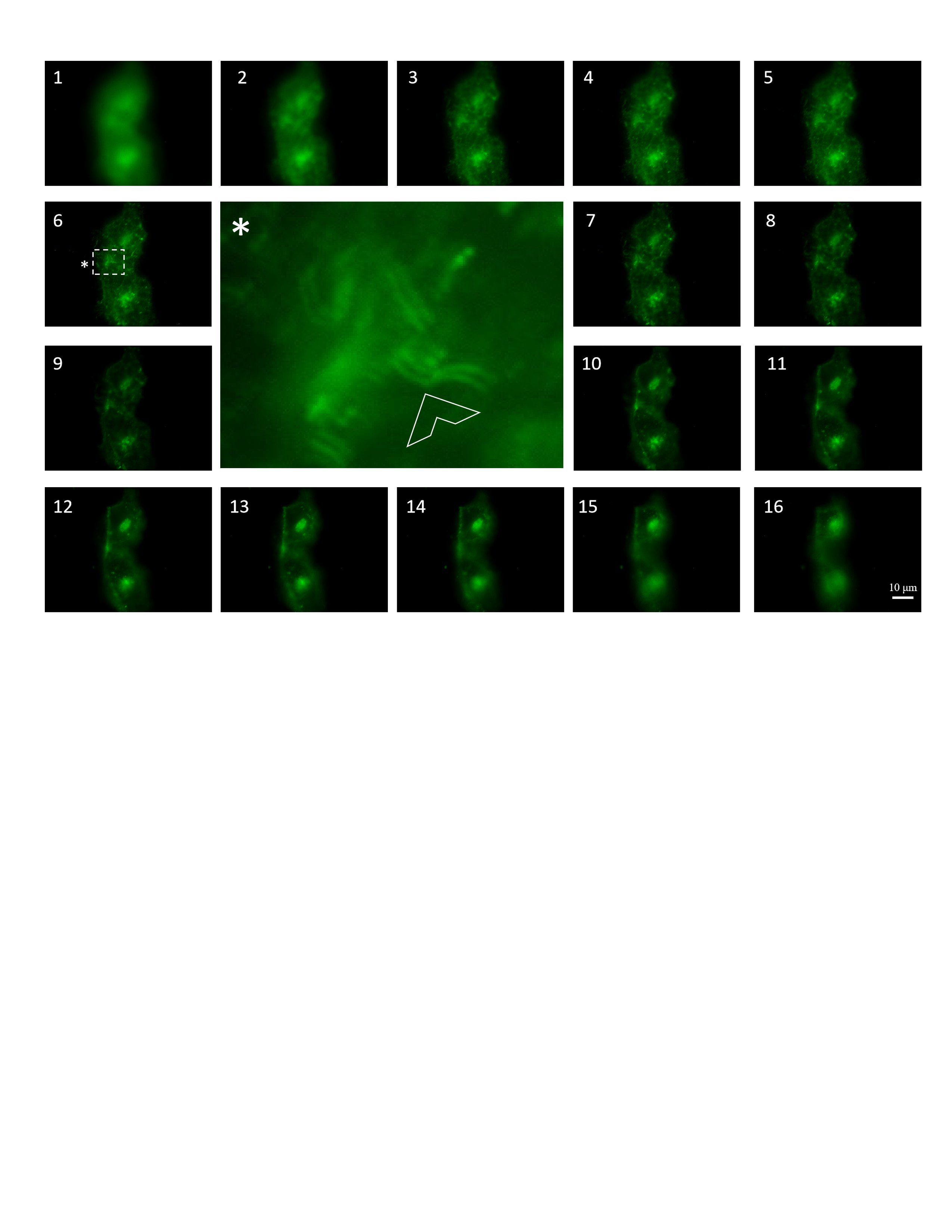

Supplement: S1 Fig — STEC were transfected with indicated EGFP-Claudin-1 for 12 h. STEC were fixed and subjected to immunofluorescence analysis to detect Claudin-1 (green). Scale bar: 10 μm. (TIF) [file ppat.1010912.s001.tif]

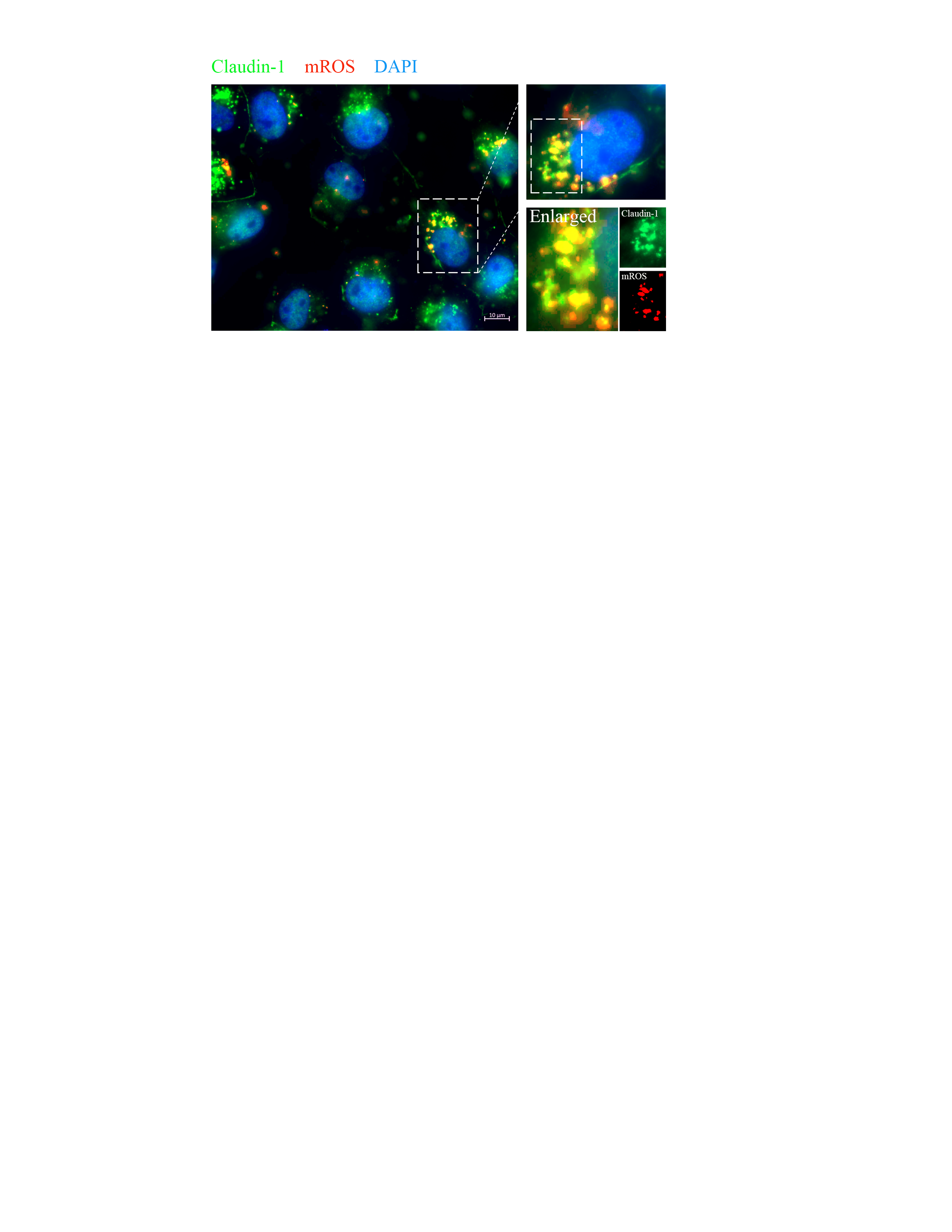

Supplement: S2 Fig — STEC infected with HPS5-SQ for 12 h were fixed and subjected to immunofluorescence analysis to detect Claudin-1 (green) and mitochondria (red) by staining Claudin-1 and mROS. Scale bar: 10 μm. (TIF) [file ppat.1010912.s002.tif]
