# Supplementary material for: Glaesserella parasuis serotype 5 breaches the porcine respiratory epithelial barrier by inducing autophagy and blocking the cell membrane Claudin-1 replenishment
Source: PLoS Pathog. 2022 Oct 13;18(10):e1010912. doi: 10.1371/journal.ppat.1010912 (PMC9595547; doi:10.1371/journal.ppat.1010912)
Supplement: S1 Table — (DOCX) [file ppat.1010912.s004.docx]

**S1 Table. The sequence of primers for the plasmid construction experiment**

| Primer | Primer Sequence (5'-3') |
| --- | --- |
| mCherry-reverse-F | GGATCCAAGCTTCTGCAG |
| mCherry-reverse-R | CTTGTACAGCTCGTCCAT |
| mCherry-EGFP-F | ATGGACGAGCTGTACAAGGCTAGCATGGTGAGCAAGGGCGAG |
| mCherry-EGFP-R | CTGCAGAAGCTTGGATCCACCTCCTCAGTTATCTAGATCCGG |
| mCherry-/mCherry-EGFP-FIS1-F | CGCGGATCCATGGAGGCCGTGCTGAAC |
| mCherry-/mCherry-EGFP-FIS1-R | CCCAAGCTTTCAGGATTTGGACTTGGATACAG |
| EGFP-Claudin-1-F | CCCAAGCTTATGGCCAACGCGGGGCTGCAG |
| EGFP-Claudin-1-R | CGCGGATCCTCACACGTAGTCTTTCCCAC |
