# Supplementary material for: Glaesserella parasuis serotype 5 breaches the porcine respiratory epithelial barrier by inducing autophagy and blocking the cell membrane Claudin-1 replenishment
Source: PLoS Pathog. 2022 Oct 13;18(10):e1010912. doi: 10.1371/journal.ppat.1010912 (PMC9595547; doi:10.1371/journal.ppat.1010912)
Supplement: S2 Table — (DOCX) [file ppat.1010912.s005.docx]

**S2 Table. The sequence of primers for quantitative RT-PCR**

| Genes | Primer Sequence (5'-3') |
| --- | --- |
| *GAPDH* Forward | CCCCTTCATTGACCTCCACT |
| *GAPDH* Reverse | TGGAAGATGGTGATGGCCTT |
| *claudin-1* Forward | TGGTCAGGCTCTCTTCACTG |
| *claudin-1* Reverse | TTGGATAGGGCCTTGGTGTT |
